# Comparison between succinylcholine and rocuronium as neuromuscular blocking agents for electroconvulsive therapy in a patient with pseudocholinesterase deficiency

**DOI:** 10.1186/s40981-015-0009-2

**Published:** 2015-08-27

**Authors:** Tomonori Takazawa, Takashi Suto, Masako Aihara, Takeshi Anzai, Tatsuo Horiuchi, Makiko H. Yamada, Yuji Kadoi, Shigeru Saito

**Affiliations:** 1 Department of Anesthesiology, Gunma University Graduate School of Medicine, 3-39-22 Showa-machi, Maebashi, 371-8511 Japan; 2 Department of Psychiatry, Gunma Prefectural Psychiatric Medical Center, 2-2374 Kunisada-machi, Isesaki, 379-2221 Japan

**Keywords:** Electroconvulsive therapy, Sugammadex, Rocuronium, Anesthesia

## Abstract

We report here the anesthetic management of a patient with schizophrenia and pseudocholinesterase deficiency. Electroconvulsive therapy was performed using succinylcholine and rocuronium as the neuromuscular blocking agents in the first seven and latter six treatments, respectively. The recovery time from muscle relaxation after succinylcholine administration was remarkably longer than that after rocuronium-sugammadex administration. Rocuronium and sugammadex appear to be useful in situations in which succinylcholine is contraindicated.

## Background

Neuromuscular blocking agents (NMBA) and intravenous anesthetics are used during modified electroconvulsive therapy (m-ECT). Indeed, bone fractures and dislocation have been reported when ECT is performed without appropriate muscle paralysis [1, 2]. The NMBA used for ECT should have a rapid onset and short duration of muscle relaxation. Therefore, previously, the choice of relaxant was limited to succinylcholine (SCC). However, succinylcholine is not always ideal, as it has some undesirable side effects, such as the risk of anaphylaxis, increasing serum potassium levels, and other cardiovascular properties [3, 4]. Since, in high doses, rocuronium (ROC) has a reasonably rapid onset and can be reversed with sugammadex, it has the potential to be used in place of succinylcholine [5–8]. We present here a patient with pseudocholinesterase deficiency in whom a series of m-ECTs was performed with either SCC or ROC.

## Case presentation

A 50-year old man (56.3 kg, 171 cm) with schizophrenia was scheduled for m-ECT. He had previously undergone ECT without anesthesia (i.e., unmodified ECT) for about 20 years, which was replaced with m-ECT because he developed a calcaneal fracture. Although preoperative blood tests indicated hypoproteinemia (total protein 6.2 g/dl, albumin 2.9 g/dl), his liver and renal functions appeared to be normal. His current medication included oral paliperidone (6 mg), aripiprazole (6 mg), chlorpromazine (100 mg, two times per day), and intramuscular injection of 50 mg risperidone.

After obtaining written informed consent, a series of m-ECTs was performed. Blood pressure, heart rate, oxygen saturation, partial pressure of carbon dioxide, and electrocardiogram were monitored during the procedure. Ventilation was assisted with a face mask using 100 % oxygen. The electrical stimulus was delivered via bifronto-temporal electrodes. The dose of propofol and NMBAs used in each treatment is shown in Table 1. Pirenzepine (10 mg) was injected to reduce oral discharge and inhibits gastric acid secretion in most cases. In the first treatment, his spontaneous breathing was not fully restored even 20 min after the stimulus. Hence, the anesthesiologist inserted a laryngeal mask just before moving to the recovery room and assisted his ventilation with a flow inflating bag for 15 min. He remained in the recovery room for about 50 min and was moved to the ward after confirming consciousness and stable respiration. In the second treatment, the first spontaneous breath appeared more than 15 min after the stimulus despite reducing the SCC dose to 20 mg. SCC was further reduced to 10 mg in the third and fourth treatment sessions; in both cases, the first spontaneous breath appeared 15 min after the stimulus. Finally, the SCC dose was reduced to 5 mg (0.09 mg/kg) in the fifth treatment session, with which the time to first spontaneous breath was shortened to 5 min.

**Table 1:** Dose of propofol and neuromuscular blocking agents, seizure duration, and duration for which the patient stayed in the operation room after the electrical stimulus

Treatment no.	Propofol (mg)	NMBA (mg)	Clinical seizure duration (s)	EEG seizure duration (s)	Time to shifting from OR (min)
1	50	SCC (40)	21	28	25
2	40	SCC (20)	24	27	22.5
3	40	SCC (10)	45	45	15
4	40	SCC (10)	25	42	15
5	40	SCC (5)	21	22	7.5
6	40	SCC (5)	28	37	7.5
7	40	SCC (6)	43	43	17
8	40	ROC (27) + SUG (250)	18	25	6.5
9	40	ROC (30) + SUG (400)	25	39	7.5
10	40	ROC (30) + SUG (400)	23	37	5.8
11	40	ROC (30) + SUG (200)	31	31	5
12	40	ROC (30) + SUG (200)	31	31	10
13	40	ROC (30) + SUG (400)	34	34	7.5

*NMBA* neuromuscular blocking agent, *SCC* succinylcholine, *ROC* rocuronium, *SUG* sugammadex

After the first series of m-ECTs, we obtained a sample of the patient’s blood to uncover the cause of the unexpected delayed recovery from muscle relaxation following administration of SCC. We also attempted to test the dose-response to SCC under neuromuscular monitoring using a train-of-four (TOF)-Watch SX (Organon, Roseland, NJ, USA) in the seventh treatment. The data acquired from the TOF-Watch was stored in a personal computer via a fiber-optic cable. The ulnar nerve was supramaximally stimulated with a square pulse of 0.2-ms duration, delivered as train-of-four (TOF) pulses at intervals of 15 s. The resulting contractions of the adductor pollicis muscles were quantified by an acceleromyographic monitor. Calibration was performed, and baseline responses were recorded after propofol administration and before muscle relaxant administration, with the neuromuscular monitoring continued until he was moved to the recovery room. For anesthesia induction, propofol followed by 2 mg SCC was administered in a titrated dose. An additional 2 mg of SCC was administered 1 min after the first administration, because the number of twitches observed (TOF count) was still four. At the same time, fasciculation of all the muscles was observed. Finally, an additional 2 mg (6 mg in total) of SCC was required 1 min after the second administration of SCC, because the TOF count was still four. T1 was assessed as being zero, 75 s after the third administration. Thereafter, the psychiatrist performed ECT. With this protocol, the first spontaneous breath was observed at 11.5 min after the ECT stimulus, and he was moved to the recovery room at 17.3 min after the stimulus. It took 19.5 min to recover to a TOF count of four. We obtained the results of the blood tests in the interval between the seventh and eighth treatments. Again, the tests indicated normal liver and renal function. However, his pseudocholinesterase level was extremely low (27 U/L, normal range 213-501 U/L), suggesting that delayed recovery from muscle relaxation following the administration of SCC was due to pseudocholinesterase deficiency. Hence, we decided to use rocuronium instead of SCC for future ECTs.

**Fig. 1:**
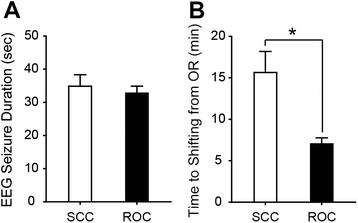
The comparison of EEG seizure duration (**a**) and time to shifting from operation room (**b**) between treatment with SCC and ROC-sugammadex. Data are mean ± SD. *P < 0.05, Mann-Whitney Rank Sum Test

In the eighth treatment session, anesthesia was induced with propofol followed by 17 mg (0.3 mg/kg) ROC under neuromuscular monitoring. An additional 10 mg of ROC (27 mg in total) was administered 2.5 min after the first administration because the TOF ratio was 0.33. ECT was performed 1.5 min after the second administration of ROC. We administered 200 mg sugammadex 1.8 min after the stimulus, resulting in appearance of the first spontaneous breath 2.3 min after the electrical stimulus. An additional dose of 50 mg of sugammadex was administered 4.8 min after the stimulus because the TOF ratio was still 0.79. He was moved to the recovery room 6.8 min after the stimulus, after confirming that the TOF ratio was 0.96. Although an abdominal CT scan was performed after the eighth treatment to explore the cause of pseudocholinesterase deficiency, no abnormalities, such as liver cirrhosis or cancer, were detected. The gastroenterologist whom we consulted did not find a definitive cause of the pseudocholinesterase deficiency, although he pointed out poor nutrition as a candidate etiology. In the ninth treatment, ECT was performed when T1 was assessed as being zero, 4 min after ROC administration. Sugammadex (400 mg) was administered 1 min after the ECT stimulus. Spontaneous respiration resumed 1 min after sugammadex administration, and bag mask ventilation was not required 0.5 min thereafter, due to the adequacy of spontaneous breathing. The TOF ratio recovered to ≥0.9, 4.5 min after the ECT stimulus. He was moved to the recovery room 1.3 min thereafter. Almost the same anesthesia protocol was followed for the tenth to thirteenth ECT sessions, although neuromuscular monitoring was not performed. In our patient, SCC was used from the first to seventh treatment, while ROC-sugammadex was used from the eighth to thirteenth sessions (Table 1). Blood tests performed after the last treatment still demonstrated low values (28 U/L) of pseudocholinesterase. We then compared the duration of electroencephalogram (EEG) seizures with SCC vs. ROC-sugammadex by Mann-Whitney Rank Sum Test (Fig. 1a) and found no statistically significant difference between them. The actual duration of seizures measured by the attending psychiatrist was also not different between the two NMBAs (data not shown). However, the duration for which the patient stayed in the operation room after the stimulus was much longer following ECT sessions with SCC as compared to those with ROC-sugammadex (Fig. 1b, *P* < 0.05, Mann-Whitney Rank Sum Test).

### Discussion

We used either SCC or ROC as the NMBA for sequential m-ECTs in a schizophrenic patient with pseudocholinesterase deficiency that was diagnosed during the course of ECTs. Although the duration of seizures was not affected by the choice of NMBA, the combination of ROC and sugammadex shortened the time to return of spontaneous respiration, and hence, the time for which the patient stayed in the operation room after the ECT. To our knowledge, this is the first report comparing the effect of SCC and ROC during m-ECT in a single patient with pseudocholinesterase deficiency.

We previously reported the potential benefit of a combination of ROC-sugammadex as an alternative to SCC for muscle relaxation during ECT [5, 6, 9]. In patients without pseudocholinesterase deficiency, 8 mg/kg sugammadex produced equally rapid recovery from ROC-induced (0.6 mg/kg) muscle relaxation compared with spontaneous recovery from 1 mg/kg SCC during ECT [6]. In the present patient, we used 200–400 mg sugammadex (5.5 ± 1.8 mg/kg) for reversal of ROC (0.52 ± 0.02 mg/kg), while the dose of SCC used was 5–40 mg (0.24 ± 0.23 mg/kg), which is much smaller than the usual dose (i.e., 1 mg/kg).

Although monitoring the TOF ratio seems to be a good method for assessing NMBA effect during ECT with non-depolarizing NMBAs, the TOF ratio has uncertain significance following administration of a single dose of SCC [10]. To overcome this problem, TOF count was used instead of TOF ratio to estimate the effect of SCC in the seventh treatment. We did not compare the time to recovery from muscle relaxation between SCC and ROC-sugammadex, because neuromuscular monitoring was only performed during some of the treatments. A definitive comparison of the NMBAs would require stringent documentation of the time to recovery of T1 to 10 and 90 % [5–7].

In this patient, the time to the first spontaneous breath after electrical stimulation in the seventh treatment was 11.5 min, while it was 2.3 and 2 min in the eighth and tenth treatments, respectively. The anesthesiologist permitted the patient to be shifted from the operation room to the recovery room once his spontaneous breathing was deemed stable enough. The duration that the patient remained in the operation room after electrical stimulation was much shorter when a combination of ROC-sugammadex was used, as shown in Fig. 1b, suggesting faster recovery from muscle relaxation with ROC-sugammadex.

Seizure duration was comparable between treatment with SCC and ROC-sugammadex, as shown in Fig. 1a. This was inconsistent with our previous report which showed longer seizure duration with ECT following ROC administration than that with SCC administration [5]. This discrepancy may be caused by the order in which NMBAs were used in this ECT series. Indeed, the number of sessions of ECT seems to affect seizure duration, because of improvement in the depressive condition with ECT. Hence, the use of ROC following that of SCC may mask the longer ECT seizure duration with ROC administration.

The relatively high cost of ROC-sugammadex reduces its advantages during ECT [11]. Indeed, in the UK, use of ROC-sugammadex (4 mg/kg) for ECT reportedly increases the cost of the drugs used during ECT by 170 times as compared to SCC administration (£125.29 vs. £0.71) [11]. In our ECT series, the average drug cost, including anesthetic and vasoactive agents for ECT, calculated from the database of the Japan Ministry of Health, Labor, and Welfare, was six times higher for treatment with ROC-sugammadex than with SCC (¥17,328 vs. ¥2,885).

Recent evidence demonstrated a risk of anaphylaxis following administration of sugammadex [12, 13], although anaphylaxis induced by muscle relaxants, including SCC and ROC, is not rare. A recent report from France demonstrated that NMBAs were the cause of perioperative anaphylaxis in more than half the cases and that SCC was the causative NMBA in more than 60 % of such cases [14]. However, it is difficult to determine which NMBA is more likely to induce anaphylaxis. This is because the frequency of use of NMBAs is unknown. Besides anaphylaxis, SCC has a range of side effects, as mentioned before. Given the varied contraindications to the use of SCC, ROC may be a safer NMBA for ECT, particularly when it is used with sugammadex.

The advantages of use of ROC-sugammadex for ECT, including fast recovery from muscle relaxation and fewer side effects, may outweigh the high cost of its usage. The superiority of ROC-sugammadex for ECT is even more obvious in subjects with pseudocholinesterase deficiency, since spontaneous recovery from muscle relaxant effects after SCC is expected to be markedly prolonged in such cases.

## Conclusions

ROC administration followed by sugammadex reversal of neuromuscular blockade seems to be better than succinylcholine for ECT, particularly in patients with pseudocholinesterase deficiency. Moreover, the usefulness of ROC and sugammadex for ECT may be extended to patients in whom the use of SCC is contraindicated, such as those with severe osteoporosis, amyotrophic lateral sclerosis, and a history of neuroleptic syndrome.

## Consent

Written informed consent was obtained from the patient for publication of this case report and any accompanying images. A copy of the written consent is available for review by the Editor-in-Chief of this journal.

## Abbreviations

ECT: electroconvulsive therapy
EEG: electroencephalogram
NMBA: neuromuscular blocking agent
ROC: rocuronium
SCC: succinylcholine
TOF: train-of-four
